# A minimal three-arm oral regimen for healthspan: mechanistic alignment with transcriptomic signals from a large parental-lifespan GWAS

**DOI:** 10.3389/fragi.2026.1856979

**Published:** 2026-08-19

**Authors:** Ngo Cheung

**Affiliations:** Independent Researcher, Hong Kong, Hong Kong SAR, China

**Keywords:** GLP-1, glutamatergic, healthspan, NAD+, NMN, plasticity, pruning

## Abstract

A large genome-wide association study of parental lifespan was reported in 2019. A later transcriptome-wide association study (TWAS) based on those summary statistics identified a set of transcriptional programs associated with longer genetically predicted survival, including increased brain NAD + salvage, especially NMNAT2, reduced glucose-stimulated insulin secretion, a shift toward synaptic pruning with less broad plasticity, and a glial pattern characterized by relatively greater microglial and lower astrocytic signatures, with only weak pan-tissue senescence signals. Building on those directional findings, this short communication proposes a minimal three-arm oral regimen with unequal evidentiary weight: first, the Cheung Glutamatergic Regimen, consisting of low-dose dextromethorphan potentiated by a CYP2D6 inhibitor together with piracetam and L-glutamine, as an exploratory adjunct aimed at preserving residual functional connectivity; second, daily nicotinamide mononucleotide and N-acetylcysteine with pulsed senolytics for NAD + salvage and senescence modulation; and third, GLP-1 receptor agonism for metabolic reprogramming. The NAD+/senescence arm is the primary mechanistic anchor, GLP-1 receptor agonism provides secondary metabolic support, and the glutamatergic arm is exploratory. Each arm targets a separate node within the pruning-plasticity-metabolic triad. The regimen is fully oral, uses conservative dosing, and draws on prior therapeutic or human-exposure data, although the proposed combination has no established safety profile. Although direct combination data are lacking and the foundational TWAS remains a preprint, the components show plausible but uneven mechanistic alignment with the TWAS signals and may justify carefully designed, safety-focused pilot evaluation.

## Introduction

Human lifespan is strongly polygenic and appears to be shaped by the cumulative effects of many common variants that act less through a generalized slowing of ageing than through protection from common age-related diseases (Timmers et al., 2019; van den Berg et al., 2017). In the largest study of its kind (Timmers et al., 2019), analyzed 1,012,240 parental lifespans and identified 12 genome-wide significant loci under common-effect-size models, including five that were novel at the time. A Bayesian extension that integrated 58 mortality risk-factor GWAS additionally highlighted associations near ABO, ZC3HC1, and IGF2R. Pathway and cell-type analyses pointed to enrichment in synaptic function, vesicle-mediated transport, fetal brain cells, and adult dorsolateral prefrontal cortex, while also showing strong links to cardiometabolic disease, smoking-related lung cancer, and dementia (Timmers et al., 2019).

Even with those findings, the biological steps linking mostly non-coding GWAS signals to organism-level survival remain unclear. This variant-to-gene problem is especially difficult in complex traits such as lifespan, where effect sizes are small and tissue context matters (Gallagher et al., 2018; Manolio et al., 2009). Transcriptome-wide association studies can partly address this gap by integrating GWAS summary statistics with tissue-specific expression quantitative trait loci data, thereby pointing to candidate genes and giving directional information about genetically predicted expression (Barbeira et al., 2018; Consortium, 2020; Gusev et al., 2016; Wainberg et al., 2019). Such associations are prioritization signals rather than proof of causal mediation and can be influenced by linkage disequilibrium, tissue choice, and prediction-model limitations (Wainberg et al., 2019).

A recent TWAS applied to the common-effect-size parental-lifespan summary statistics from Timmers et al. (Timmers et al., 2019) across 49 GTEx tissues identified several consistent transcriptional programs (Cheung, 2026b). The analysis remains a preprint and has not yet undergone independent peer review. Its directional signals are broadly compatible with independent longevity GWAS and trans-omic analyses that implicate metabolic, inflammatory, and age-related disease biology (Deelen et al., 2019; Pilling et al., 2017; Perrot et al., 2021). The strongest positive signal was upregulation of the KEGG nicotinate and nicotinamide metabolism pathway, most evident in brain caudate, cortex, and frontal cortex, and driven largely by NMNAT2 together with other NAD + salvage genes. By contrast, genes involved in glucose-stimulated insulin secretion showed the strongest negative enrichment. Long-term potentiation and synaptic plasticity programs were downregulated, whereas synapse pruning and microglial signatures were modestly increased and astrocyte signatures were decreased. Cellular senescence pathways showed little coherent pan-tissue direction, and mitochondrial programs appeared mixed rather than uniformly shifted in one direction (Cheung, 2026b; Perrot et al., 2021).

Taken together, these findings suggest that common variants associated with longer life may act by supporting neuronal maintenance, moderating metabolic signaling, refining synaptic architecture, and permitting selective cellular clearance. Interventions aimed at only one pathway have generally produced modest gains in human healthspan, possibly because they touch only one part of a broader network (López-Otín et al., 2023). A more coherent approach may therefore involve simultaneous, non-redundant targeting of several nodes within the pruning-plasticity-metabolic framework.

This manuscript outlines a minimal three-arm oral regimen intended to engage several of the transcriptional programs identified by the TWAS. The stack includes the Cheung Glutamatergic Regimen as an exploratory adjunct aimed at preserving residual functional connectivity, a senescence-modulator arm for NAD + salvage and cellular maintenance, and GLP-1 receptor agonism for metabolic reprogramming. The components were selected for mechanistic complementarity, oral administration, practical feasibility, and prior therapeutic or human-exposure data. The aim is not to claim lifespan extension, but to propose a practical hypothesis-generating multi-pathway framework that may deserve formal evaluation in light of the genomic findings.

## Methods

**FIGURE 1:**
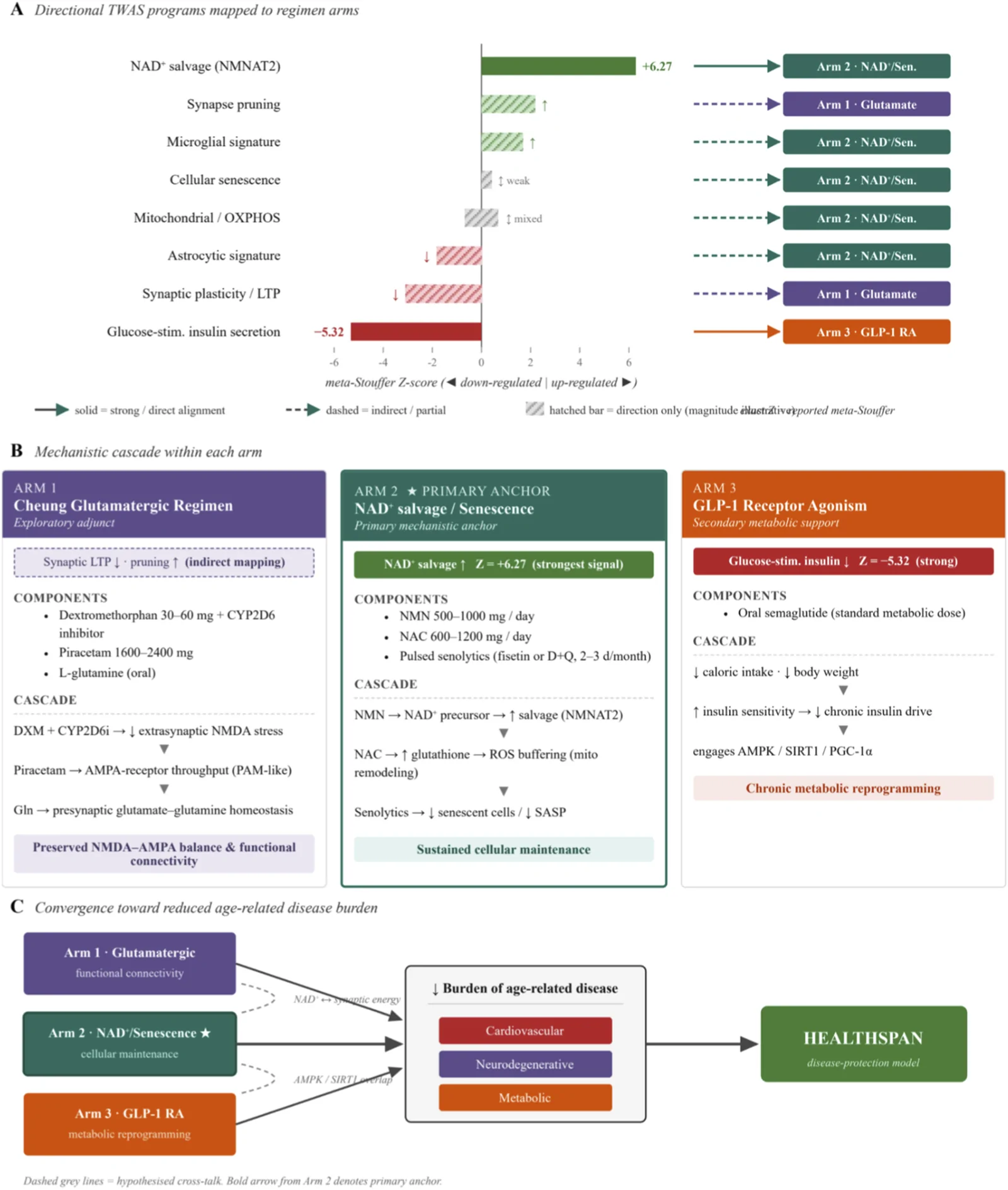
Mechanistic alignment of the proposed three-arm oral regimen with transcriptomic signals from a tissue-resolved TWAS of parental lifespan (Timmers et al., 2019 GWAS; n = 1,012,240). **(A)** Horizontal bars depict meta-Stouffer Z-scores for TWAS-identified programs (positive = upregulated in longer genetically predicted lifespan; negative = downregulated). Arrows map each program to the pharmacological arm with greatest mechanistic relevance; solid arrows indicate strong alignment, dashed arrows indicate indirect or partial alignment. **(B)** Mechanistic cascades within each arm. Arm 1 (Cheung Glutamatergic Regimen) targets NMDA–AMPA balance and presynaptic glutamate homeostasis; Arm 2 supplies NAD^+^ precursor, antioxidant buffering, and intermittent senescent-cell clearance; Arm 3 reduces chronic insulin drive while engaging AMPK/SIRT1 pathways. Arm 2 is the primary mechanistic anchor, Arm 3 provides secondary metabolic support, and Arm 1 is exploratory because its mapping to the lifespan TWAS is indirect. Coloured TWAS badges indicate the direction and strength of the primary transcriptomic signal addressed by each arm. **(C)** Convergence model showing how the three arms may jointly reduce the burden of age-related disease (cardiovascular, neurodegenerative, metabolic) and thereby support healthspan. Dashed lines indicate hypothesised cross-talk (e.g., NAD^+^ supporting synaptic energy demands; AMPK/SIRT1 overlap between Arms 2 and 3). The overall framework is consistent with the finding that common lifespan-associated variants appear to act primarily through disease protection rather than generalised ageing deceleration. Abbreviations: DXM, dextromethorphan; CYP2D6i, CYP2D6 inhibitor; Gln, L-glutamine; PAM, positive allosteric modulator; NMN, nicotinamide mononucleotide; NAC, N-acetyl-L-cysteine; SASP, senescence-associated secretory phenotype; GLP-1 RA, glucagon-like peptide-1 receptor agonist; LTP, long-term potentiation; BDNF, brain-derived neurotrophic factor; ROS, reactive oxygen species.

The TWAS used the common-effect-size parental-lifespan summary statistics from Timmers et al. (Timmers et al., 2019), based on 1,012,240 parents, together with GTEx v8 eQTL weights across 49 tissues (Consortium, 2020) (Figure 1). Genetically predicted gene expression was imputed using FUSION (Gusev et al., 2016). Gene-set enrichment was assessed with Stouffer’s combined Z-score, mean and median Z_lifespan, Wilcoxon rank-sum testing, and empirical permutation p-values derived from 10,000 iterations. Seven prespecified categories were examined: NAD/sirtuin/nicotinamide metabolism, cellular senescence, synaptic pruning/plasticity, insulin/GLP-1/cAMP signaling, mitochondrial/oxidative phosphorylation, apoptosis, and glial markers, including microglial, astrocytic, and oligodendrocytic markers. Driver genes were identified by ranking individual Z_lifespan values within enriched sets.

A secondary multi-filter sensitivity analysis additionally applied pooled-FDR and leave-three-out criteria to assess the robustness of the gene-set signals.

The three-arm regimen was then assembled by mapping those TWAS signals to existing oral agents with prior therapeutic or human-exposure data and plausible mechanistic relevance. Selection required alignment with at least one major TWAS program, a primary mechanism that did not simply duplicate the others, conservative dosing to reduce risk, and practical feasibility in outpatient settings. No invasive routes and no novel compounds were included.

## Results and discussion

In the primary analysis, the TWAS showed that increased brain NAD + salvage was the strongest positive signal, with KEGG nicotinate and nicotinamide metabolism reaching a meta Stouffer Z of 6.268. The signal was most prominent in cortical tissue and was driven by NMNAT2, with Z_lifespan of 8.0312, together with PNP, ENPP1, and NADSYN1. Although the KEGG nicotinate and nicotinamide metabolism pathway showed the strongest positive meta-Stouffer Z (6.268) and was driven by NMNAT2, the signal did not survive the more stringent pooled-FDR or leave-three-out multi-filter criteria applied in secondary analyses. The NAD + arm is therefore motivated primarily by the directional TWAS result and by mechanistic plausibility, rather than by uniformly robust statistical support across all sensitivity filters. This limitation is acknowledged, and the regimen is framed as hypothesis-generating. Because TWAS prioritization does not establish causality, formal colocalization and Mendelian-randomization analyses will be needed before assigning causal status to NMNAT2 or related genes (Wainberg et al., 2019; Giambartolomei et al., 2014; Zuber et al., 2022). This pattern was not a blanket increase across all NAD-related biology: genes involved specifically in NAD + salvage and metabolism tended to be upregulated, whereas broader NAD + -binding and biosynthesis modules tended to move in the negative direction. The second strongest directional result was reduced insulin-secretory signaling in response to glucose, with a meta Stouffer Z of −5.3186, especially in tibial nerve, skeletal muscle, thyroid, and cerebellum. Synaptic plasticity and long-term potentiation programs were broadly negative, whereas synapse pruning showed modest positive enrichment. Glial markers suggested a shift toward microglia and away from astrocytes. Cellular senescence signals were weak, inconsistent, and bimodal across genes. Mitochondrial programs were also heterogeneous, with some respiratory genes positively associated and others strongly negative, fitting a model of remodeling rather than uniform suppression (Cheung, 2026b).

The proposed three-arm regimen was assembled with unequal weight reflecting the strength of TWAS alignment. The NAD+/senescence arm is the primary mechanistic anchor; GLP-1 receptor agonism provides secondary metabolic support; and the glutamatergic arm is retained only as an exploratory functional adjunct.

The first arm, the Cheung Glutamatergic Regimen, combines low-dose dextromethorphan at 30–60 mg daily, potentiated by a CYP2D6 inhibitor, with piracetam at 1,600 to 2,400 mg daily and oral L-glutamine. Although the TWAS suggested a tilt toward pruning rather than broadly enhanced plasticity, this regimen may still support cognitive function by modulating glutamatergic tone, limiting extrasynaptic NMDA stress, and supporting AMPA throughput (Cheung, 2026a; Cheung, 2026c). This arm does not map directly onto the TWAS enrichment for pruning over plasticity and is therefore given lower priority than the other two arms. It is retained as an exploratory adjunct because an observational cohort associated DXM exposure with lower dementia incidence and a randomized clinical trial of dextromethorphan-quinidine reported symptomatic benefit for Alzheimer disease agitation; neither line of evidence establishes dementia prevention or disease modification (Chen et al., 2022; Cummings et al., 2015). Glutamine supplementation has also shown neuroprotective effects in transgenic models and a protective signal against Alzheimer’s disease in Mendelian randomization analysis (Adams, 2020; Son et al., 2023). In this framework, the glutamatergic arm may indirectly fit the TWAS pattern by helping preserve functional synaptic connectivity without broadly driving plasticity.

As the primary mechanistic anchor, the second arm targets cellular maintenance and includes daily nicotinamide mononucleotide at 500 to 1,000 mg and N-acetylcysteine at 600 to 1,200 mg, together with pulsed senolytics, either fisetin or dasatinib plus quercetin, given only for two to three consecutive days per month. NMN aligns directly with the strongest positive TWAS signal, namely, increased nicotinate and nicotinamide metabolism and higher NMNAT2 expression in brain tissue (Cheung, 2026b). However, the human evidence supporting NMN remains short-term and does not establish healthspan benefit; randomized evidence has not shown significant improvements in glucose or lipid outcomes (Song et al., 2023; Fukamizu et al., 2022; Yi et al., 2023; Chen et al., 2024). NAC may help buffer oxidative stress in a context where mitochondrial programs appeared mixed and remodeled rather than simply up- or downregulated (Bradlow et al., 2022). Pulsed senolytics are intended to reduce senescence-associated secretory phenotype burden without imposing continuous suppression. Human evidence for intermittent senolytic treatment remains preliminary and is largely confined to small pilot studies of dasatinib plus quercetin or related senolytic strategies (Hickson et al., 2019; Justice et al., 2019; Gonzales et al., 2023), whereas evidence for fisetin remains predominantly preclinical (Yousefzadeh et al., 2018).

The third arm uses GLP-1 receptor agonism, specifically oral semaglutide at standard metabolic doses. This choice aligns with the strongest negative TWAS program, namely, downregulation of glucose-stimulated insulin secretion, while still preserving incretin responsiveness (Liu, 2024). Although acute GLP-1 receptor agonism stimulates glucose-dependent insulin secretion, chronic therapy can improve insulin sensitivity and reduce caloric intake and body weight, potentially lowering overall insulin requirements, while engaging AMPK/SIRT1/PGC-1α pathways that overlap conceptually with NAD + biology (Liu, 2024; Smits et al., 2016; Huang et al., 2025). This chronic metabolic reprogramming is more congruent with the TWAS signal of reduced glucose-stimulated insulin secretion than acute pharmacology alone would suggest; nonetheless, the alignment remains indirect. Clinical studies in adults with overweight or obesity, including people without diabetes, provide relevant but indication-specific human exposure data for semaglutide (Wilding et al., 2021; Wilding et al., 2022; Rubino et al., 2022; Alissou et al., 2026).

These three components were selected for complementarity rather than redundancy. The glutamatergic arm is intended to support functional synaptic maintenance, the senescence-modulator arm directly addresses the dominant NAD + salvage signature, and GLP-1 receptor agonism reinforces the reduced insulin-drive signal. All components are oral and have some prior therapeutic or human-exposure data, but the proposed combination has no established safety profile (Cheung, 2026a; Cheung, 2026c; Chen et al., 2022; Cummings et al., 2015; Song et al., 2023; Fukamizu et al., 2022; Yi et al., 2023; Hickson et al., 2019; Justice et al., 2019; Gonzales et al., 2023). Because semaglutide access and pricing vary substantially by setting, no reliable monthly cost estimate is provided.

Safety considerations. Safety considerations differ across the three arms and must be addressed explicitly before any pilot work (Table 1). For the glutamatergic arm, CYP2D6 inhibition—particularly with bupropion as the inhibitor—can materially alter exposure to CYP2D6 substrates, and dextromethorphan has been implicated in serotonin toxicity when combined with serotonergic agents (Kotlyar et al., 2005; Schwartz et al., 2008). Conservative dosing (30–60 mg dextromethorphan) and avoidance of concomitant strong serotonergic drugs are therefore mandatory.

**TABLE 1:** Safety considerations for the proposed three-arm oral regimen.

Arm	Key agents and dosing	Principal safety considerations	Practical precautions
Arm 1 Glutamatergic (exploratory)	Dextromethorphan 30–60 mg daily + CYP2D6 inhibitor + piracetam 1 600–2 400 mg daily + L-glutamine	CYP2D6 inhibition substantially elevates dextromethorphan exposure Documented risk of serotonin toxicity when combined with serotonergic agents	Strict conservative dosing Avoid concomitant strong serotonergic drugs Monitor for early signs of serotonin excess
Arm 2 NAD+/senescence (primary)	NMN 500–1 000 mg daily + NAC 600–1 200 mg daily + pulsed senolytic (fisetin or dasatinib + quercetin, 2–3 consecutive days per month)	Short-term NMN trials (up to ∼1 250 mg) report acceptable tolerability; long-term safety data remain limited Human evidence for intermittent senolytics is preliminary and confined to small pilot studies Continuous senolytic exposure is deliberately avoided	Prefer intermittent (pulsed) senolytic schedules only Restrict use to supervised settings until longer-term safety data accumulate
Arm 3 GLP-1 receptor agonism (secondary)	Oral semaglutide at standard metabolic doses	Long-term risk–benefit balance is less clear in metabolically healthy or non-diabetic individuals without a clear metabolic indication Gastrointestinal adverse effects are common Absolute lean-mass reduction can accompany large weight loss (net functional effect remains uncertain) Weight regain and reversal of cardiometabolic gains after discontinuation are well documented	Use only under close clinical monitoring Explicit discussion of gastrointestinal effects, lean-mass change, and rebound risk required before initiation

The three components have individual prior therapeutic or human-exposure data, but the proposed combination has no established safety profile. No component should be self-administered as a healthspan intervention outside appropriately supervised clinical or research settings.

For the NAD+/senolytic arm, short-term human trials of NMN, including doses up to approximately 1,250 mg daily, have reported acceptable tolerability (Fukamizu et al., 2022; Yi et al., 2023), yet long-term safety data remain limited and randomized evidence has not demonstrated consistent metabolic benefit (Song et al., 2023; Chen et al., 2024). Pulsed senolytics such as fisetin or dasatinib plus quercetin have only preliminary human evidence (Yousefzadeh et al., 2018; Hickson et al., 2019; Justice et al., 2019; Gonzales et al., 2023); continuous administration is deliberately avoided.

For the GLP-1 arm, the long-term risk–benefit balance is less clear in metabolically healthy or otherwise non-diabetic individuals without a clear metabolic indication. Gastrointestinal adverse effects are common, and large weight loss can be accompanied by an absolute reduction in lean mass even when the proportion of lean mass increases. Recent data have also reported preserved lean mass and improved muscle function in some settings, so the net muscle effect remains uncertain (Wilding et al., 2021; Rubino et al., 2022; Alissou et al., 2026). Weight regain and reversal of several cardiometabolic improvements after discontinuation have been documented (Wilding et al., 2022). Standard metabolic doses should be used only under close monitoring and after explicit discussion of these trade-offs.

No component should be self-administered as a healthspan intervention outside appropriately supervised clinical or research settings.

This proposed regimen is not presented as proof of lifespan extension. The TWAS that supplies the primary directional signals remains a preprint and has not yet undergone independent peer review; the regimen is therefore presented as hypothesis-generating rather than as a direct translation of validated causal genes. At most, it represents a practical candidate framework that appears to align mechanistically with several transcriptomic programs associated with longer genetically predicted lifespan. Any benefit, if it exists, would most plausibly come through lowering the burden of common age-related disease, particularly cardiometabolic and neurodegenerative conditions, rather than through a generalized slowing of ageing itself (Timmers et al., 2019). The glutamatergic component requires especially cautious interpretation because its support of synaptic function does not map directly onto the TWAS signal favoring pruning over broad plasticity. No study has tested the full combination, and any synergy remains hypothetical.

The most appropriate next step would be small open-label pilot studies focused on safety, tolerability, adherence, and target engagement, including markers such as NAD + metabolites, insulin dynamics, inflammatory indices, and cognitive or synaptic biomarkers. If those data were favorable, larger randomized trials in genetically or biomarker-stratified groups could then examine healthspan-relevant outcomes more directly. Further work should also include colocalization and Mendelian randomization analyses for top driver genes and replication in non-European populations.

In summary, this minimal three-arm oral regimen offers an outpatient-compatible framework that appears to align, with unequal strength, with several directional transcriptional programs linked to longer genetically predicted lifespan. The idea remains preliminary and rests on directional transcriptomic signals of variable statistical robustness; it may nonetheless offer one practical route for translating genomic observations into testable, multi-pathway healthspan strategies.

## Data Availability

All data generated or analysed during this study are included in this published article and its supplementary information files.
